# Remote vs. In-person Delivery of LearningRx One-on-One Cognitive Training During the COVID-19 Pandemic: A Non-inferiority Study

**DOI:** 10.3389/fpsyg.2021.749898

**Published:** 2021-10-29

**Authors:** Amy Lawson Moore, Terissa Michele Miller, Christina Ledbetter

**Affiliations:** ^1^Department of Psychology, Gibson Institute of Cognitive Research, Colorado Springs, CO, United States; ^2^Department of Neurosurgery, Louisiana State University Health Sciences Center, Shreveport, LA, United States

**Keywords:** cognitive training, teletherapy, virtual therapy, brain training, LearningRx

## Abstract

The COVID-19 pandemic challenged in-person delivery of cognitive training. Some clinics pivoted to remote delivery for those impacted by lockdowns, illness, or fear of exposure to the virus. However, it was unknown if remote delivery using teleconferencing technology was as effective as in-person delivery. The current study compared the outcomes of remote delivery to in-person delivery of ThinkRx cognitive training during 2020. The sample included 381 child and adult clients from 18 cognitive training centers. One group (*n* = 178, mean age = 12.3) received traditional in-person delivery of cognitive training. The second group (*n* = 203, mean age = 11.7) received remote delivery of one-on-one cognitive training *via* Zoom teleconferencing. Each client was assessed before and after the intervention using the Woodcock Johnson IV Tests of Cognitive Abilities. Clients completed an average of 112 h of cognitive training delivered by a clinician in 90-min sessions 3 or 4 days per week. Paired samples *t*-tests revealed significant differences from pretest to post-test across all constructs for both groups. After Bonferroni correction, MANOVA revealed no significant difference in changes scores between the two intervention groups on any of the subtests. With very small effect sizes, linear regression analyses indicated that age was a significant predictor of change in working memory and processing speed for the in-person group, and a significant predictor of change in overall IQ score for the teletherapy group. Non-inferiority analyses indicated remote delivery is not inferior to in-person delivery on the primary outcome measure of overall IQ score along with processing speed, fluid reasoning, long-term memory, and visual processing. Although in-person training results were slightly higher than remote training results, the current study reveals remote delivery of cognitive training during COVID-19 was a viable alternative to in-person delivery of cognitive training with little practical differences based on the age of client.

## Introduction

When the COVID-19 outbreak caused much of the United States to lockdown, many providers of in-person cognitive training were challenged to continue delivering the intervention through teleconferencing technology (Owens et al., 2020; Lee et al., 2021). Educators and clinicians scrambled as well to adopt remote delivery options, and evidence-based research began to trickle in on the non-inferiority of these interventions (Doraiswamy et al., 2020; Koonin et al., 2020; Monaghesh and Hajizadeh, 2020; Wosik et al., 2020).

There is over a decade of research substantiating the effectiveness of remote delivery of mental health interventions (Andersson and Cuijpers, 2009; Andersson et al., 2011; Herbert et al., 2016). Remote delivery is needed now more than ever for offering more timely diagnoses and interventions (Smith et al., 2017; Lee et al., 2021), increasing the reach of treatment options for patients in underserved settings (Nelson and Patton, 2016) or with distance, health and/or time constraints (Acierno et al., 2017; Ratzliff and Sunderji, 2018; Owens et al., 2020). Research confirms comparable outcomes for remote and in-person delivery of a wide variety of psychological and cognitive treatments (Backhaus et al., 2012; Bashshur et al., 2016), reveals corresponding user satisfaction for both methods (Cox et al., 2017; Müller et al., 2017), and affirms greater cost-effectiveness in most cases (Hubley et al., 2016).

Additionally, the literature on cognitive training is rife with research on independent-user, digital-based cognitive training apps, games, and devices (Bonnechère et al., 2020; Irazoki et al., 2020; Owens et al., 2020). Yet there are very few published articles regarding remote-delivery of cognitive training interventions as telehealth, described by Gately et al. (2019) as a “live, synchronous encounter that employs a videoconferencing software.” Thus the current study attempts to address this gap regarding the potential non-inferiority of remote delivery of one-on-one cognitive training vs. in-person one-on-one cognitive training using the LearningRx methodology.

The efficacy and effectiveness of the LearningRx cognitive training methodology has been previously demonstrated in multiple studies on various populations including children with learning struggles (Carpenter et al., 2016; Gibson et al., 2015; Jedlicka, 2017; Moore et al., 2019a), children with ADHD (Moore et al., 2018), adolescents and adults with brain injury (Ledbetter et al., 2017; Moore et al., 2020) and adults with age-related cognitive decline (James et al., 2019; Moore et al., 2019b). Results have included statistically and clinically significant changes in working memory, long-term memory, processing speed, fluid reasoning, visual processing, auditory processing, and Word Attack skills as well as reported improvements in self-esteem, self-discipline, cooperative behaviors, mood, perseverance, activities of daily living, and reduced oppositional behaviors and academic struggles. Changes in brain network connectivity, changes in the Default Mode Network (DMN), and correlations between changes in IQ score and white matter integrity have also been documented following LearningRx cognitive training (James et al., 2019; Moore et al., 2020). Because cognition is complex, cognitive training should match that complexity by targeting the multiple cognitive skills used for thinking and learning. Brain training applications and programs that only target one or two skills such as working memory or attention, fail to address the variety of constructs required for complex thought. The LearningRx methodology is designed to target multiple overlapping cognitive constructs aligned with the Cattel-Horn-Carrol theory of cognition, the most widely recognized intelligence theory and the one in which most intelligence tests are based. This comprehensive nature coupled with human delivery of the LearningRx methodology yields an advantage over digital game applications.

Although the research results on LearningRx have been robust, it was unknown if remote delivery of the intervention was as effective as the traditional in-person delivery model. Therefore, the current study compared the outcomes of clients who received remote training of LearningRx cognitive training during the COVID-19 pandemic with clients who continued to receive in-person training during the same time period. This is an important question to answer given the inherent benefits of the LearningRx methods and the transfer effects beyond the trained tasks that have been widely documented thus far. These transfer effects situate the one-on-one delivery of cognitive training at an advantage over digital applications and continue to be the holy grail of cognitive training research. If these benefits can extend to the remote environment, the program could be made available to people who are experience barriers to access including a global pandemic, severe weather, illness or injury, or geographical distance between potential clients and a cognitive training center.

## Materials and Methods

### Sample

The sample was comprised of *n* = 381 clients from 18 cognitive training clinics, including 353 children and 28 adults. Inclusion criteria included clients at least 4 years of age, completion of a cognitive training program in 2020, and completion of both pre and post intervention assessments with the Woodcock Johnson IV Tests of Cognitive Abilities. Records of children under 4 years of age were excluded. Group 1 (*n* = 178) received traditional in-person delivery of cognitive training and will be referred to henceforth as the “In-Person” group. Group composition was 39% female (*n* = 69) and 61% male (*n* = 109) with a mean age of 12.3 (SD = 8.6). Diagnoses included ADHD (*n* = 45), autism spectrum disorder (*n* = 11), dyslexia/reading disability (*n* = 38), speech and language disorder (*n* = 15), and traumatic brain injury (*n* = 1). There were 165 children (mean age = 10.5) and 13 adults (mean age = 35.1). Group 2 (*n* = 203) received remote delivery of cognitive training *via* teleconferencing and will be referred to henceforth as the “Remote” group. Group composition was 42% female (*n* = 86) and 58% male (*n* = 117) with a mean age of 11.7 (SD = 6.5). There were 188 children (mean age = 10.4) and 15 adults (mean age = 28.1). Diagnoses included ADHD (*n* = 59), autism spectrum disorder (*n* = 10), dyslexia/reading disability (*n* = 47), speech and language disorder (*n* = 18), and traumatic brain injury (*n* = 7). Table 1 shows the distribution of age and sex by group. All records were included in the analysis after determining a missing data percentage of only 1.3%. Little and Rubin (2002) indicate that data loss <5% will result in the same conclusion as if the dataset were complete.

**Table 1:** Distribution of age and sex by group.

	**In-person group**	**Remote group**
Children		
Age 4–7	30	35
Age 8–12	87	98
Age 13–17	48	56
Male	103	109
Female	62	79
Total children	165	188
Adults		
Age 18–29	7	11
Age 30–49	3	2
Age 50–69	2	2
Age 70+	1	0
Male	6	8
Female	7	7
Total adults	13	15
Total	178	203

### Procedures

De-identified digital records were collected from 18 cognitive training clinics that provided the LearningRx cognitive training program during the COVID-19 pandemic. Individual records included quantitative test results from an assessment performed before and after the intervention using the Woodcock Johnson IV Tests of Cognitive Abilities, and qualitative results from an exit survey administered by each training center at the conclusion of the intervention. Ethics approval for the acquisition and analysis of the records was granted by the Institutional Review Board (IRB) at Gibson Institute of Cognitive Research in accordance with exempt research Category 4 of 45 CFR 46.101(b)(4). Permission to use the records for research was granted in writing by clients age 18 and over or by parents of clients under the age of 18 in accordance with the Declaration of Helsinki.

### Intervention

#### In-Person Cognitive Training

**Figure 1:**
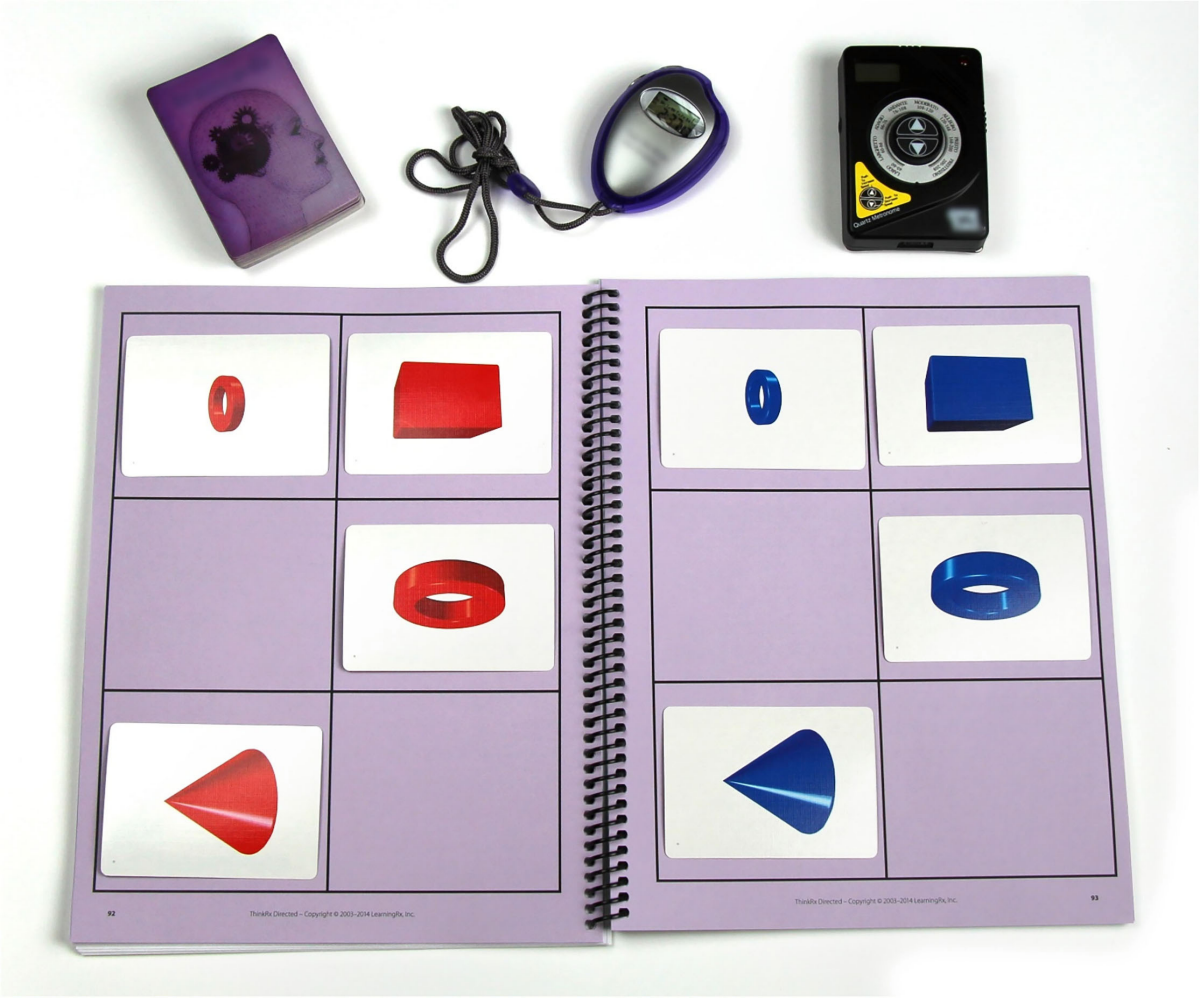
Example of a training task.

The core LearningRx program, called ThinkRx, is a 230-page curriculum with more than 1,000 variations of 23 basic training tasks sequenced by difficulty and complexity and paced by a metronome beat or stopwatch. Based on the Cattell-Horn-Carrol (CHC) theory of a multiple construct view of cognition, the training procedures target multiple cognitive skills including various aspects of working memory, long-term memory, processing speed, visual and auditory processing, fluid reasoning, and attention (Schneider and McGrew, 2018). No task is trained in isolation, however. Instead, training tasks target overlapping cognitive skills. For example, the memory training task illustrated in Figure 1 targets visual working memory, processing speed, sustained attention, visual processing, and rapid task switching. The trainer creates a pattern of five cards with similarly-sized shapes on one side of the workboard, allows the client to study the pattern for 3 sec, then covers the pattern and asks the client to reproduce it on their side of the workboard. Paced by a metronome, the client is also asked to count by three's on every other beat while completing the task. This is a description of just one variation of this training task. It can be delivered in 34 different ways. For example, a more difficult variation utilizes 8 cards with shapes of different sizes.

**Figure 2:**
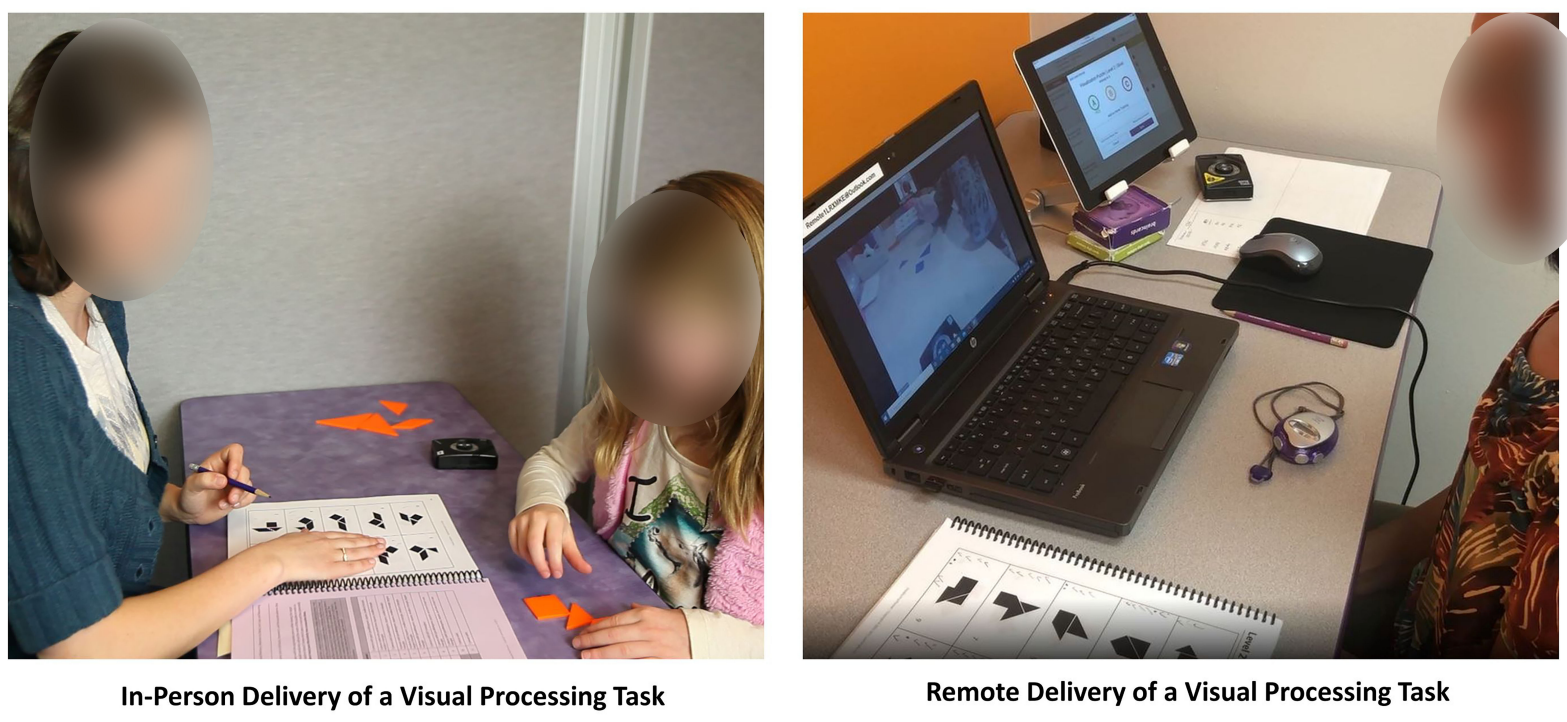
In-person vs. remote delivery of an example training task.

The trainer utilizes hands-on materials including work boards, shape and number cards, blocks, Tangrams, and speeded activity worksheets to deliver the program. Hands-on tasks may be loaded by the trainer with additional verbal tasks to train rapid task switching ability. In the traditional delivery method, clients attend their training sessions sitting one-on-one across a table from their cognitive trainer (see Figure 2). The trainer paces the session, manages frustration levels, provides dynamic feedback and motivating verbal persuasion, adds deliberate distractions to mimic real world learning and working environments, and helps the client apply what's learned in training to tasks outside of the training environment. This human-delivery format is a departure from the ubiquitous digital “brain training” methods described in much of the field's literature. In addition, this model of interaction is illustrative of Bandura (1994) self-efficacy theory by providing all four sources of self-efficacy for learning including modeling, mastery experiences, verbal persuasion, and guided management of the physiological response to stress. In the current study, the in-person training group received the intervention in the traditional one-on-on setting.

Clients completed an average of 112 h of cognitive training delivered by a cognitive trainer in 90-min sessions 3 or 4 days per week. A complete description of the training tasks, the number of variations per task, and the cognitive constructs targeted by each task was previously published in Carpenter et al. (2016). In brief, Table 2 illustrates the constructs targeted by the program, the number of training tasks that target each construct, and the number of variations for each training task.

**Table 2:** Cognitive constructs targeted by the LearningRx training program.

**Construct trained**	**# of Tasks**	**# of Variations**
Auditory analysis	5	53
Auditory blending	1	11
Auditory discrimination	5	57
Auditory processing	4	48
Auditory segmenting	1	12
Comprehension	5	155
Divided attention	11	314
Executive processing	4	154
Fluid reasoning	5	159
Long-term memory	4	147
Computation	6	197
Processing speed	12	380
Saccadic fixation	2	88
Selective attention	5	189
Sensory motor integration	7	156
Sequential processing	1	17
Short-term memory	5	134
Simultaneous processing	10	317
Sustained attention	11	359
Visual processing	9	273
Visual discrimination	3	118
Visual manipulation	5	176
Visualization	10	271
Visual span	4	147
Working memory	12	431

#### Remote Training

To approximate the in-person training experience, clients in the remote training group were sent an identical set of hands-on materials, metronome, and an external webcam on a tripod that projected their workspace for the trainer during each videoconferencing session. The trainer used both tangible materials visible on the webcam as well as pdf versions of the workboards for the client to see projected on their monitor. The interactions and time in training were identical between the groups. A video that illustrates two training procedures delivered through both methods is available at https://youtu.be/Xb65X2HVf_E. Figure 2 illustrates the difference between in-person and remote delivery of an example training task.

For both training delivery methods, the curriculum is adaptable for different ages and skills levels. Although the training tasks are the same for everyone, older clients may master the early, easier variations within the first couple of training sessions. Their sessions would then focus on the more challenging variations of the tasks. For all ages, more time is focused on the tasks in which the struggle is greatest with very little time spent on tasks that are easy for each individual. Therefore, it's plausible that some clients spend more time on working memory tasks, for example, if that's a construct in which they are most deficient. Other clients may have stronger working memory and thus their training session may be more focused on building competency in other skills. The great number of task variations enables the trainer to individualize the protocols to match the client's needs and skills.

### Outcome Measures

#### Woodcock Johnson IV Tests of Cognitive Abilities (WJ IV)

Each participant was administered the Woodcock Johnson IV Tests of Cognitive Abilities (Schrank et al., 2014) immediately before and after the intervention. The WJ IV measures individual cognitive constructs as well as a composite IQ score, in comparison to an age-based normative group. For the current study, we selected the overall IQ score as well as subtests used by LearningRx centers to report pretest to post-test change The subtests were chosen based on their ability to measure key constructs in the Cattel-Horn-Carroll (CHC) theory of cognition (Schneider and McGrew, 2018) which is the theory in which most major IQ test are based. The LearningRx program is also based on CHC theory which recognizes the complex overlap of multiple broad and narrow cognitive skills. A description of each measure is below.

##### Overall IQ *Score*

IQ score was measured using the General Intellectual Ability composite which represents a measure of overall intelligence and cognitive performance. It is a weighted composite of Tests 1–7 in the core WJIV battery. This composite score has a median reliability of 0.97.

##### Working *Memory*

Working memory was measured by the Numbers Reversed test, a classic task where the examinee is asked to hold an increasingly complex set of numbers in awareness and then orally reverse the sequence. This test has a median reliability of 0.88.

##### Long-term *Memory*

Long-term memory was assessed using the Visual-Auditory Learning subtest where the examinee memorizes a set of symbols and their corresponding words before being asked to “read” sentences of symbols based on remembering the associated words. This test has a median reliability of 0.97.

##### Processing *Speed*

Processing speed was measured by the Letter-Pattern Matching subtest which requires the examinee to make visual symbol discriminations among a series of increasingly more complex letter patterns in a 3-min time limit. This test has a reliability of 0.91 for ages 7–11 and ages 26–79 and a reliability of 0.88 for ages 14–17.

##### Visual Processing

Visual processing was measured by the 2-part Visualization subtest which captures performance in visual feature detection and the mental rotation of objects. The first part asks the examinee to identify two or three individual pieces that combine to form a completed shape. The second part asks the examinee to identify rotated block configurations that match the target configuration. This test has a median reliability of 0.85.

##### Auditory *Processing*

Auditory processing was measured by the 3-part Phonological Processing subtest. The first part asks the examinee to listen to a sound and then produce a word that contains that sound either at the beginning, in the middle, or at the end as prompted by the examiner. The second part assesses word recall ability, and the third part requires the examinee to substitute a sound in a word to produce a new word. This test has a median reliability of 0.84.

##### Sustained *Attention*

Sustained attention was measured using the Pair Cancellation subtest which asks the examinee to find and mark repeating and increasingly more complex patterns of shapes within a 3-min time frame. This test has a reliability of 0.89 for ages 7–11 and ages 14–17 and a reliability of 0.95 for ages 26–79.

##### Fluid Reasoning

Fluid Reasoning was measured by the Concept Formation subtest which targets inductive logic by requiring the examinee to derive a rule from each stimulus set of shapes with various characteristics. This test has a median reliability of 0.93.

#### Qualitative Exit Surveys

When clients at LearningRx centers finish a cognitive training program, they are asked to complete an exit survey about their experience. The open-ended question on the survey asks them to “Please share with us the changes you have seen as a result of the LearningRx training.”

### Data Analyses

#### Quantitative Data Analyses

##### Within Group Change

De-identified data were transferred from EXCEL to SPSS Version 27 for analyses. To evaluate the significance of pretest to post-test change within each intervention group, we conducted paired samples *t*-tests on the pre and post WJIV standard scores. To control for multiple comparisons, we applied a Bonferroni correction to the significance threshold making the adjusted alpha *p* < 0.006. To determine the magnitude of significance, we calculated effect sizes using Cohen's *d* defined with Cohen (1988) general guidance of small (0.2), medium (0.5), or large (0.8) effects.

##### Between Group Differences

To evaluate any baseline differences between the two intervention groups, we conducted a multivariate analysis of variance (MANOVA) on all pretest scores. Then, to evaluate differences in change scores between the two intervention groups, we conducted a multivariate analysis of variance (MANOVA) with the dependent variables being the difference between pretest and post-test standard scores for each construct, or change scores. To control for multiple comparisons, we applied a Bonferroni correction to the significance threshold making the adjusted alpha *p* < 0.006. To determine the magnitude of significance, we annotated effect sizes using multivariate eta square defined as small (0.01), medium (0.06), and large (0.14 or higher).

##### Non-inferiority

To determine if the remote training method of delivery was not inferior to the in-person method of delivery, we used the non-inferiority margin as described by Walker (2019). First, we determined what percentage of the traditional gains were necessary to preserve in the new delivery method. Although a preserved fraction of 50% is common in non-inferiority trials (Althunian et al., 2017), we opted for a more rigorous threshold by choosing a more conservative and clinically relevant 75% preserved fraction of the effect based on the results from prior controlled studies on the in-person delivery method along with the clinical judgement of 6 cognitive training experts we polled. We used two previous controlled trials of LearningRx (Gibson et al., 2015; Carpenter et al., 2016) plus the current study to determine the pooled mean change across constructs weighted by sample size for traditional in-person delivery (M = 12.5 standard points) and then applied the 75% threshold to determine the delta for the current trial (Δ = −3.75). To meet the 75% preserved fraction of the effect seen in the in-person group and to conclude non-inferiority of the remote training method, the lower end of the 95% confidence interval (CI) around the mean difference in each score change could not be lower than the non-inferiority margin of −3.75. We calculated the 95% CI for each outcome variable using the following formula:

(μ_new_ – μ_control_) ± 1.96 $\sqrt{\frac{\sigma^{2}new}{\text{n}\;\text{new}}+\frac{\sigma^{2}control}{\text{n}\;\text{control}}}$

Where “new” represents remote and “control” represents in-person, and 1.96 is the z value required for a one-tailed alpha of 0.025.

##### Differences by Age

To determine if age was a significant predictor of change on any of the constructs measured, we conducted hierarchical linear regression analyses using age in months as the predictor variable and change in standard score on each measure as the outcome variable. We included the pretest score in each model as a covariate predictor block. To control for multiple comparisons, we applied a Bonferroni correction to the significance threshold making the adjusted alpha *p* < 0.006. To determine the magnitude of significance of the overall model, we annotated effect sizes using R square. To determine how much of the variance in each dependent variable is explained by age after controlling for pretest scores, we squared the Beta coefficient and converted to a percentage.

#### Qualitative Data Analyses

We used a standard grounded theory 3-step process of coding, analysis, and development of themes (Kiger and Varpio, 2020) to evaluate the written statements made by the 304 of the 381 participants who completed the exit survey at the end of the intervention. This inductive analysis ensures that themes emerge from the data instead of creating pre-determined themes of expected adherence (Charmaz and Thornberg, 2020). During the thematic analysis, the qualitative researcher was blind to group identification. The analysis began with line-by-line reading of comments without coding or categorization, followed by a second review while note taking to ascertain the wide variety of responses. Then, data were coded at the phrase level and then themes were evaluated and clarified by the research team. Finally, the responses were unblinded to group identification for comparison of themes between the two groups.

## Results

### Quantitative Results

#### Within Group Change

After Bonferroni correction for multiple comparisons to an alpha of *p* < 0.006, paired samples *t*-tests revealed significant differences from pretest to post-test across constructs for both groups with medium to large effect sizes represented by Cohen's *d* as shown in Table 3. For the In-Person Group, the largest change was seen in auditory processing followed by overall IQ score, sustained attention, and fluid reasoning. For the Remote Group, the largest change was also seen in auditory processing along with overall IQ score, followed by fluid reasoning and sustained attention.

**Table 3:** Paired sample *t*-tests of pretest to post-test change by group.

**In-Person**					**Remote**			
**WJ4**	**Pre (SD)**	**Post (SD)**	***p***	***d***	**Pre (SD)**	**Post (SD)**	***p***	***d***
IQ	92.9 (16.2)	104.7 (16.8)	0.000	1.19	90.9 (16.6)	100.9 (16.1)	0.000	1.07
WM	94.6 (15.8)	104.6 (15.4)	0.000	0.69	92.8 (16.8)	100.9 (16.3)	0.000	0.51
LTM	99.4 (12.7)	108.2 (13.5)	0.000	0.78	98.9 (12.9)	107.1 (14.4)	0.000	0.77
PS	93.2 (14.0)	100.8 (12.5)	0.000	0.74	91.9 (15.2)	98.4 (15.2)	0.000	0.54
VP	100.9 (13.8)	108.4 (13.7)	0.000	0.63	99.2 (14.3)	105.9 (14.1)	0.000	0.60
AP	89.2 (15.8)	104.1 (15.2)	0.000	1.23	88.1 (16.3)	100.3 (15.6)	0.000	1.07
Attn	92.1 (13.5)	103.6 (14.3)	0.000	0.95	92.1 (14.3)	101.2 (13.7)	0.000	0.79
FR	100.6 (16.2)	110.6 (16.6)	0.000	0.93	98.1 (17.2)	107.9 (17.4)	0.000	0.84

*Significance level set at Bonferroni-corrected alpha p < 0.006. d, Cohen's d effect size; WM, working memory; LTM, long-term memory; PS, processing speed; VP, visual processing; AP, auditory processing; Attn, sustained attention; FR, fluid reasoning; IQ, IQ score composite*.

#### Between Group Differences

A MANOVA on pretest measures indicated there were no statistically significant differences between the groups at baseline on any of the pretest scores: IQ score (*p* = 0.190), processing speed (*p* = 0.299), auditory processing (*p* = 0.391), visual processing (*p* = 0.211), fluid reasoning (*p* = 0.164), working memory (*p* = 0.291), long-term memory (*p* = 0.669), or attention (*p* = 0.948). As shown in Table 4, the MANOVA on change scores revealed no significant difference in changes scores between the two intervention groups on any of the subtests after Bonferroni correction for multiple comparisons to an alpha of *p* < 0.006 (Wilk's Lambda = 0.97, *F* = 1.32, *p* = 0.23, η^2^ = 0.031). The In-Person group saw slightly larger change scores than the Remote Group on all constructs measured, differing the most in auditory processing with a 2.5 point difference in standard score change.

**Table 4:** MANOVA of standard score change by group.

**Woodcock Johnson IV Measure**	**In-Person Group** **Mean Change Score (SD)**	**Remote Group** **Mean Change Score (SD)**	**F**	**p**	**η^2^**
IQ Score	11.7 (9.8)	10.0 (9.3)	1.8	0.178	0.005
Working Memory (WM)	10.0 (14.4)	8.1 (15.9)	1.2	0.273	0.004
Long-term Memory (LTM)	8.8 (11.2)	8.2 (10.6)	0.64	0.426	0.002
Processing Speed (PS)	7.6 (10.3)	6.5 (12.1)	0.98	0.323	0.003
Visual Processing (VP)	7.6 (11.9)	6.7 (11.1)	0.57	0.449	0.002
Auditory Processing (AP)	14.8 (12.0)	12.3 (11.5)	5.58	0.019	0.016
Attention (Attn)	11.4 (12.0)	9.1 (11.5)	6.18	0.013	0.018
Fluid Reasoning (FR)	10.0 (10.7)	9.7 (11.5)	0.001	0.971	0.000

*Significance level set at Bonferroni-corrected alpha p < 0.006*.

#### Non-inferiority

**Figure 3:**
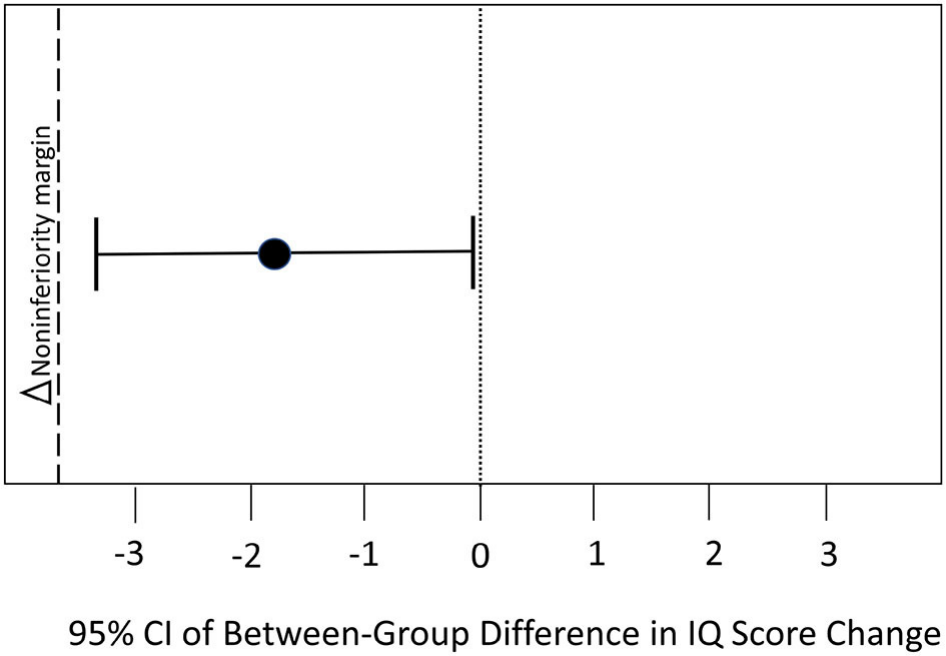
Results of non-inferiority testing on overall IQ score.

To meet the 75% preserved fraction of the effect seen in the in-person group and to conclude non-inferiority of the remote training method, the lower end of the 95% confidence interval around the mean difference in each score change could not be lower than the non-inferiority margin. IQ score is a composite index representing performance on seven cognitive constructs and serves as a indicator of overall cognitive function. We chose to use the change in overall IQ score as the primary outcome by which to report the non-inferiority analysis. The non-inferiority margin is −3.75 points. Therefore, to meet the 75% preserved fraction of the effect seen in the in-person group, the lower end of the 95% confidence interval around the mean difference in IQ score change could not be lower than −3.75. Results show the 95% CI of the mean between-group difference in IQ score change was −0.226 to −3.40, falling above the non-inferiority margin. Therefore, the remote delivery method is not inferior to the in-person delivery method in changing overall IQ score. Figure 3 illustrates this analysis.

Using the same threshold, we evaluated non-inferiority for each of the remaining outcome variables. To meet the 75% preserved fraction of the effect seen in the in-person group and to conclude non-inferiority of the remote training method on each construct, the lower end of the 95% confidence interval around the mean difference in each score change could not be lower than the non-inferiority margin of −3.75. Non-inferiority was demonstrated for fluid reasoning (95% CI = −0.60, 1.91), processing speed (95% CI = −2.20, 1.16), visual processing (95% CI = −1.80, 1.43), and long-term memory (95% CI = −1.50, −3.39), but was inconclusive for the outcomes of auditory processing (95% CI = −6.80, −1.92), working memory (95% CI = −4.40, 0.58), and attention (95% CI = −4.60, 0.02). That is, part of the confidence interval fell below the non-inferiority margin for those latter constructs.

#### Differences by Age

As shown in Table 5, linear regression analyses indicated that after controlling for pretest scores, age was not a significant predictor of change in any of the cognitive test scores from pretest to post-test for the remote training group. For the In-Person Group, age was a significant predictor after controlling for pretest scores in working memory (β = 0.190, *p* = 0.004, R^2^ = 0.273) indicating that for every year in age, the change in test score increased by 0.19 points from pretest to post-test. However, only 3.6% of the variance in scores can be explained by age. Age was also a significant predictor of change in sustained attention for the in-person group (β = 0.270, *p* = 0.000, R^2^ = 0.213) indicating that for every year in age, the change in score increased by 0.27 points from pretest to post-test. However, only 7.2% of the variance in sustained attention scores can be explained by age.

**Table 5:** Regression analysis of age as a predictor of outcomes by group.

	**In person group**				**Remote group**			
	***B***	***t***	***p***	***R^**2**^***	***B***	***t***	***p***	***R^**2**^***
IQ score	0.186	2.4	0.016	0.091	0.169	2.4	0.018	0.128
WM	0.190	2.9	0.004^*^	0.273	−0.072	1.2	0.239	0.261
LTM	0.114	1.6	0.107	0.149	0.010	0.142	0.888	0.073
PS	0.059	0.89	0.372	0.263	−0.036	0.55	0.583	0.159
VP	0.054	0.78	0.434	0.198	−0.044	0.64	0.525	0.167
AP	0.087	1.2	0.214	0.190	0.065	0.99	0.319	0.176
Attn	0.270	3.9	0.000^*^	0.213	0.043	0.67	0.504	0.208
FR	−0.033	0.45	0.654	0.089	−0.104	1.5	0.124	0.114

* *Significant at Bonferroni-corrected alpha p < 0.006. B, Beta coefficient of slope; R^2^, effect size; WM, working memory; LTM, long-term memory; PS, processing speed; VP, visual processing; AP, auditory processing; Attn, sustained attention; FR, fluid reasoning; IQ, General Intellectual Ability composite*.

### Qualitative Results

The analysis of qualitative responses to the exit interview uncovered three predominant themes (cognitive changes, academic changes, behavioral changes) with 12 subthemes. *Cognitive changes* included the subthemes of cognitive test scores, memory, logic and reasoning, processing, and attention. *Academic changes* had subthemes of math skills, reading/writing, and grades/school performance. *Behavioral changes* included subthemes of confidence, personal responsibility, social skills, and mood/outlook.

**Figure 4:**
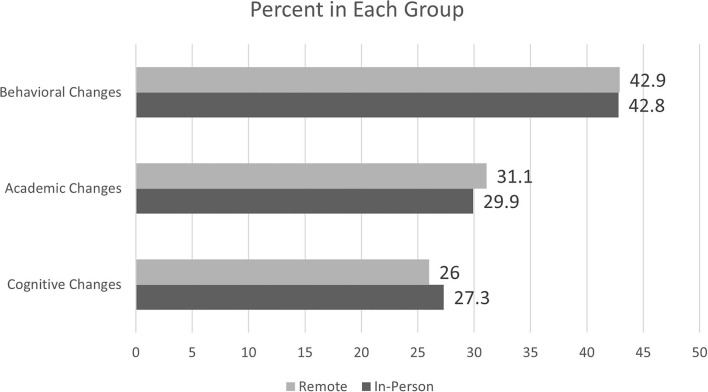
Percent of each group reporting change in each theme.

Results indicate very little difference between the two groups in regard to the three primary themes. In the In-Person Training Group, 27.3% of the total comments were *cognitive changes*, 29.9% were *academic changes*, and 42.8% were *behavioral changes*. Similarly, in the Remote Training Group, 26.0% of their responses were *cognitive changes*, 31.1% were *academic changes*, and 42.9% were *behavioral changes*. These comparisons are illustrated in Figure 4.

Participants who commented about *cognitive changes* wrote things like, “He has gotten stronger with puzzles, logic, and his memory is better,” “…better focus to complete homework,” “…results of the tests showed that she significantly improved,” and “…improved attention to detail.” Most of the remarks about *academic changes* referenced reading and writing, which included statements such as, “…increased reading skills, increased reading fluency,” “…a renewed enjoyment of reading,” “Math skills and reading comprehension have grown,” and “Her ability to read has and will open up so many doors for her.” The largest percentage of responses were about *behavioral changes*, with a majority of remarks about confidence such as, “Her self-esteem has soared,” “[He] has become much more confident in himself and his abilities,” “She is happier,” “Our son has become more independent in completing his homework,” and “My daughter has shown tremendous growth in her courage to try new things.” Most respondents were parents/guardians of cognitive training clients, although 7 were adult (over age 21) clients themselves. The responses from these adult participants did not differ from other remarks except for one which specified impact within the workplace: “…improved long and short term memory and visual processing when at work.”

There was a total of 304 research participants who responded to the exit survey, with 79 who gave a general comment with no uniquely identifiable theme. These responses included statements like, “trainers were great,” “we believe in the program,” and “they helped our son.” Thus, the thematic analysis outcome was evaluated based on 225 respondents who offered specific remarks, with a total of 271 unique comments from those who trained in-person, and 273 unique comments from those who trained remotely. Finally, about 6% of the overall responses in each group were negative, including dissatisfaction with cost, time commitment, results, or the process such as, “I was hoping to see more improvements in school and grades,” “It could have been better adjusted for age,” and “It was not a bad experience, but I think the program is overpriced and requires a huge time commitment.”

## Discussion

The aim of the current study was to address a gap in the literature regarding the potential non-inferiority of remote delivery of one-on-one cognitive training vs. in-person one-on-one cognitive training. The efficacy of the LearningRx in-person cognitive training methodology had been previously demonstrated in multiple studies (Carpenter et al., 2016; Moore et al., 2019a,b, 2018), but it was unknown prior to this study if remote delivery of the intervention was as effective as the traditional in-person delivery model. We found statistically significant changes across all constructs measured (working memory, long-term memory, processing speed, visual and auditory processing, attention, reasoning, and overall IQ score) for both intervention groups with robust effect sizes. We also found no statistically significant differences between the two delivery methods on any of the measures. Inferiority analyses demonstrated that remote delivery is not inferior to in-person delivery for the primary outcome measure of overall IQ score and for the individual constructs of processing speed, fluid reasoning, long-term memory, and visual processing. Inferiority analyses was inconclusive for auditory processing, working memory, and attention since part of the 95% confidence interval fell below the margin. Finally, we evaluated the qualitative outcomes and found three themes consistent in both groups: changes in cognition, changes in academic skills, and changes in behavior and psychosocial functioning.

Because the results were so similar between the two methods of delivering this intervention, the current study suggests that remote delivery is a feasible alternative to in-person delivery of the same intervention which enables more widespread access to this cognitive training program. The implication of this benefit should not be understated given the potential barriers to in-person access including another global pandemic, severe weather, illness or injury, and geographical distance between potential clients and a cognitive training center. These barriers are significant given that the LearningRx program is only available in-person in 70 locations, most in highly populated areas. Rural areas of the country lack access to a training center within driving distance. In addition, our post-COVID society is cautious about face-to-face contact and a virtual alternative to this intervention may help to allay anxiety about potential exposure to illnesses. Further, remote access enables continuity of care more easily should a client be traveling or experiencing a mild illness or injury. Although additional research is necessary to evaluate equivalence of the two methods given the results of the inferiority analyses, the finding of statistically significant pretest to post-test changes on all constructs in both delivery methods lends additional evidence to the existing body of research on the effectiveness of the LearningRx training program. Future research should evaluate motivation as it is historically a key contributor to performance. Adding quantitative psychosocial outcome measures would also strengthen future research and continue building the case for transfer effects beyond the trained tasks and cognitive measures.

Although there were no statistically significant differences in changes on cognitive test scores between the two groups, it is worth noting that the In-Person Training group did achieve slightly higher change scores than the Remote Training group on all of the constructs measured and non-inferiority could not be confirmed for three of the individual constructs. There are several possible reasons for this trend including Zoom fatigue for the Remote Training participants who were already spending their day learning and working virtually during the pandemic. Another possibility is the strength of engagement may have been less in a virtual environment vs. an in-person environment. Finally, the degree of external stimuli is greater in the traditional, in-person method of delivering this intervention in a busy open-concept training room at LearningRx centers. Having up to 15 training stations in the same room forces participants to focus on only the relevant stimuli and tasks while tuning out the noise and motions surrounding them. This increased intensity may give the In-Person Training group the slight edge over the Remote Training group participants who wore headphones and trained in quieter surroundings.

The strengths of the current study include a robust sample size with diverse ages, giving us a solid understanding of the feasibility of adapting the in-person delivery method across age groups. Another strength of the study is the ecological validity of evaluating real-world outcomes. The data were extracted from actual cognitive training clients who were living through a global pandemic—a quintessential scenario in which pivoting to remote interventions is necessary. A final strength of the study is the inclusion of qualitative data to evaluate transfer effects of both delivery methods. Indeed, the themes uncovered through the qualitative analysis were consistent with themes uncovered in prior research on the LearningRx cognitive training methods (Ledbetter et al., 2017; Moore et al., 2018, 2019a,b, 2020).

There are a few limitations worth noting, however. First, the groups were pre-existing and not randomly assigned. Although the groups were similar in demographics and the ecological validity of the findings is supported by the evaluation of pre-existing groups, the lack of random assignment does make the study design less robust. Next, the small number of adult participants reduces the ability to completely generalize the results to an adult population without further study. However, the regression analysis did not suggest that the results differ with much practical significance by age. The final limitation is that we did not quantify changes in constructs beyond cognition such as motivation, depression and mood, or self-efficacy. However, the addition of the qualitative data did allow us to identify trends in transfer effects and increase the value of the study to the field.

## Conclusions

The current study analyzed real-world data from an adaptation of a well-established in-person cognitive training method to a teletherapy delivery method during the COVID-19 pandemic in 2020. Results indicated that remote delivery was not only feasible but showed similar results as in-person training with little practical differences based on the age of client. These findings support the use of remote delivery of the LearningRx one-on-on cognitive training intervention and suggest the barriers to access of the traditional in-person delivery can be reduced through remote availability.

## Data Availability Statement

The raw data supporting the conclusions of this article will be made available by the authors, without undue reservation.

## Ethics Statement

The studies involving human participants were reviewed and approved by Gibson Institute of Cognitive Research IRB. Written informed consent to participate in this study was provided by the participants' legal guardian/next of kin.

## Author Contributions

AM acted in the capacity of primary investigator, conducted the quantitative data analysis, and drafted the manuscript. CL acted as co-PI, created the study design, oversaw data acquisition and storage, and edited the manuscript. TM drafted the literature review, conducted the qualitative data analysis, and edited the manuscript. All authors contributed to the article and approved the submitted version.

## Funding

This research was paid for with internal research funds at Gibson Institute of Cognitive Research.

## Conflict of Interest

AM is employed by the cognitive training organization whose results are evaluated in the current study and volunteers on the board of directors of the 501c3 non-profit research institute founded by the creator of the intervention used in the current study, but has no financial interest in the company or the outcomes of the research. TM is contracted by the 501c3 non-profit research institute founded by the creator of the intervention used in the current study but has no financial interest in the organization or the outcomes of the research. CL volunteers on the scientific advisory board of the cognitive training organization whose results are evaluated in the current study and on the board of directors of the 501c3 non-profit research institute founded by the creator of the intervention used in the current study but receives no financial compensation of any kind for either role.

## Publisher's Note

All claims expressed in this article are solely those of the authors and do not necessarily represent those of their affiliated organizations, or those of the publisher, the editors and the reviewers. Any product that may be evaluated in this article, or claim that may be made by its manufacturer, is not guaranteed or endorsed by the publisher.
